# Spiracular fluttering increases oxygen uptake

**DOI:** 10.1371/journal.pone.0232450

**Published:** 2020-05-20

**Authors:** Sean D. Lawley, Michael C. Reed, H. Frederik Nijhout

**Affiliations:** 1 Department of Mathematics, University of Utah, Salt Lake City, UT, United States of America; 2 Department of Mathematics, Duke University, Durham, NC, United States of America; 3 Department of Biology, Duke University, Durham, NC, United States of America; USDA Agricultural Research Service, UNITED STATES

## Abstract

Many insects show discontinuous respiration with three phases, open, closed, and fluttering, in which the spiracles open and close rapidly. The relative durations of the three phases and the rate of fluttering during the flutter phase vary for individual insects depending on developmental stage and activity, vary between insects of the same species, and vary even more between different species. We studied how the rate of oxygen uptake during the flutter phase depends on the rate of fluttering. Using a mathematical model of oxygen diffusion in the insect tracheal system, we derive a formula for oxygen uptake during the flutter phase and how it depends on the length of the tracheal system, percentage of time open during the flutter phase, and the flutter rate. Surprisingly, our results show that an insect can have its spiracles closed a high percentage of time during the flutter phase and yet receive almost as much oxygen as if the spiracles were always open, provided the spiracles open and close rapidly. We investigate the respiratory gain due to fluttering for four specific insects. Our formula shows that respiratory gain increases with body size and with increased rate of fluttering. Therefore, insects can regulate their rate of oxygen uptake by varying the rate of fluttering while keeping the spiracles closed during a large fraction of the time during the flutter phase. We also use a mathematical model to show that water loss is approximately proportional to the percentage of time the spiracles are open. Thus, insects can achieve both high oxygen intake and low water loss by keeping the spiracles closed most of the time and fluttering while open, thereby decoupling the challenge of preventing water loss from the challenge of obtaining adequate oxygen uptake.

## 1 Introduction

Insects have an efficient mechanism of respiration in which atmospheric air is taken directly to every cell in the body via a system of tracheae. Tracheae open to the outside air via a set of openings called spiracles. The tracheal system is lined with the same cuticle that makes up the exoskeleton and forms a progressively more finely branching system of air-filled tubes. The terminal ends of the tracheae, called tracheoles, deliver oxygen individually to every cell in the body [1]. The tracheal system is also the principal route for the removal of carbon dioxide produced by metabolism, and is also, potentially, a major avenue of water loss. The spiracles have muscular mechanisms that can close the tracheal system, and the respiratory system is so efficient that many insects can keep their spiracles closed for long periods of time to conserve water. When insects are forced to keep their spiracles open, by exposing them to 5% carbon dioxide, their rate of water loss increases two- to ten-fold; *Rhodnius prolixus*, an otherwise drought resistant species, dies within 3 days [1, 2].

Many insects exhibit a pattern of discontinuous respiration in which periods that spiracles are open alternate with periods during which they are closed and periods during which the spiracles open and close rapidly, a phenomenon known as fluttering [3–5]. The open phase is often very brief and is associated with a rapid release of carbon dioxide [6, 7], whereas oxygen uptake occurs during the fluttering phase. The patterns of spiracle opening, closing, and fluttering vary for individual insects depending on developmental stage and activity, vary between insects of the same species, and vary even more between different species [8–10].

There have been many hypotheses about the reasons for discontinuous gas exchange. For instance, closure of spiracles reduces entry of pathogens into the respiratory system [11]. Another function might be to reduce the concentration of oxygen in the trachea system to minimize the effects of oxygen toxicity [12]. Closure of the spiracles can serve to steepen the oxygen gradient in the tracheal system which would improve gas exchange in oxygen poor habitats [13]. The oldest and most famous hypothesis is that the closure of the spiracles prevents respiratory water loss [6]. These and other hypotheses are reviewed in [5].

Quantitative analysis of diffusion in insect tracheae dates back to the early work of Krogh [14], and there have been many recent studies that address specific quantitative questions. For example, Snyder et al. [15] emphasized the importance of tracheal dimensions as adaptive adjustments in insect gas exchange. Förster and Hetz [16] experimentally altered levels of oxygen and carbon dioxide inside the tracheal system and noted when the insects opened and closed their spiracles and when the insects started fluttering. Simelane et al. [17] considered partial pressures, allowed the volume of the tracheal tube to change based on pressure, and noted that during periods of flutter, the interior pressure is slightly negative compared to atmospheric pressure. The authors conclude that “the change in CO2 elimination is directly related to the level of oxygen uptake into the trachea.” Grieshaber and Terblanche [18] created a biologically realistic pure diffusion model using interacting oxygen and carbon dioxide control systems and suggested that a control system with two interacting feedback loops could generate the observed discontinuous gas exchange patterns.

In this paper, we address two aspects of the deep and interesting physics and physiology questions associated with discontinuous gas exchange. First, how does oxygen uptake during the flutter phase depend on the speed of fluttering? How does it compare to the oxygen uptake if the spiracles are always open? We determine how the uptake during the flutter phase depends on the flutter rate, *r*, the diffusion coefficient of oxygen, *D*, the tracheal length, *L*, and the proportion of time the spiracles are open, *p*. Let *U*_open_ be the oxygen uptake per unit time if the spiracle is always open, and let *U*_flutter_ be the oxygen uptake per unit time during the flutter phase. We define the *flutter factor*, to be the ratio *f* = *U*_flutter_/*U*_open_, i.e.

$$U_{\text{flutter}}=fU_{\text{open}}.$$

Of course, *f* will depend on the parameters *p*, *r*, *L*, *D*. Using mathematical analysis [19], we derived a formula for *f*,

$$f=f\left(p,r,L,D\right)={\left[1+\frac{1-p}{p}\sqrt{\frac{D}{L^{2}r}}\text{tanh}(\sqrt{\frac{L^{2}r}{D}})\right]}^{-1}.$$ (1)

This formula allows us to calculate the dependence of *f* on the parameters *p*, *r*, *L*, *D*. The factor *f* is always between 0 and 1 and describes how much the flutter phase decreases oxygen uptake compared to *U*_open_.

Surprisingly, our results show that an insect can have its spiracles closed a high percentage of time during the flutter phase (small *p*) and yet receive almost as much oxygen as if the spiracles were always open, provided the spiracles open and close rapidly (large *r*). What this means is that insects can control their oxygen uptake during the flutter phase by varying the rate of fluttering, and, indeed, it is known that some insects change their rate of fluttering as environmental conditions or oxygen needs change [7, 20–22]. The mathematical model for oxygen uptake while fluttering is given in Section 2 and the results in Section 3 where individual insects are considered.

Secondly, we address the question of water loss. Since the rate of fluttering has a large effect on oxygen uptake, it is natural to ask how the rate of fluttering affects water loss. In Section 4 we present a mathematical model for water loss while fluttering. Analysis of the model shows that the water loss is depends on the transfer coefficient from tissues to the tracheal tubes. When this coefficient is small there is little water loss, and when the coefficient is high the water loss is proportional to the percentage of time the spiracles are open. Thus, by keeping the percentage of time open small and by fluttering quickly, insects can achieve both high oxygen uptake *and* low water loss.

The mathematical models described in Sections 2 and 4 make a number of simplifying assumptions about the biology (addressed in the Discussion and the Appendix) that enable us to compute the (relatively) elementary formulas for oxygen uptake and water loss. Without these simplifying assumptions the formulas would be much more complicated or accessible only through machine computation. However, the general principle that we have discovered, that the insect can control the rate of oxygen uptake in the flutter phase by varying the rate of flutter, would still be true. It is known that not only diffusion but also convection plays a role in gas exchange [8, 23, 24]. In the Appendix, we extend the mathematical model from Section 2 to include convection and show that the inclusion of convection does not alter the basic results of Section 3.

## 2 The mathematical model

We consider a single spiracle opening to a trachea that branches profusely and ends in a set of tracheoles. For simplicity, we ignore the fact that that there are tracheal trunks that connect the tracheae of adjacent spiracles. We assume that the tracheae branch in such a way that the cross-sectional area remains constant, an assumption based on studies of several types of insects (see [14] for *Cossus* larva, [25] for *Aphelocheirus*, [26] for *Rhodnius*, and [27] for *Aeshna* and *Schistocerca gregaria*). We assume that the total length of the tracheal tubes that connect the spiracle to a terminal tracheole is the same for each tracheole.

As tracheoles are the primary site of oxygen absorption and the tracheae are of little significance in this respect [27], we assume that oxygen is absorbed only at tracheoles and not along the sides of the tracheae. Since the diameter of even the finest branches of the tracheae is much larger than that of an oxygen molecule, we assume that oxygen freely diffuses in the tracheae [27]. Although it is known that convection (advection) also plays a role in oxygen exchange [8, 23, 24], we ignore convection here for simplicity, and consider convection in the Appendix. As it is known that open and closed durations fluctuate during the flutter phase [7, 28], we take them to be randomly distributed with a mean that is determined from experimental data. The specific distribution we choose is exponential, but choosing another distribution (or letting the open and closed durations be nonrandom) would have little effect on our results.

Since tracheael tubes are thin, we treat each tube in the tracheal network as one-dimensional. Furthermore, since the total cross-sectional area of the tracheae is constant along the tracheal network, it follows that branching has no effect on the oxygen flux to the tracheoles [29]. In particular, it is enough to consider diffusion in an interval of length *L*, where *L* is the total length of the tracheal tubes that connect the spiracle to a tracheole.

In our model, the tracheoles are at *x* = 0 and the spiracle is at *x* = *L* and *c*(*x*, *t*) denotes the oxygen concentration in the trachea at *x* at time *t*. Then, *c*(*x*, *t*) satisfies the diffusion equation and boundary conditions

$$\frac{\partial c}{\partial t}\left(x,t\right)=D\frac{\partial^{2}c}{\partial x^{2}}\left(x,t\right),\;x\in \left(0,L\right),\;t>0,$$ (2)

c(0,t)=0, (3)

c(L,t)=A,whenspiracleopen, (4)

$$\frac{\partial c}{\partial x}\left(L,t\right)=0,\;\text{when}\;\text{spiracle}\;\text{closed}.$$ (5)

Notice that the partial differential equation always remains the same, but the boundary condition at *x* = *L* switches according to the opening and closing of the spiracle.

Since oxygen is absorbed at the tracheoles, we use the absorbing boundary condition (3) at *x* = 0. When the spiracle is open, the oxygen concentration at the spiracle (4) equals the ambient oxygen concentration *A*. When the spiracle is closed, we impose a no flux boundary condition (5) at the spiracle.

If the spiracle were always open, then the steady state oxygen concentration in the trachea would be the linear function

$$c_{\text{open}}\left(x\right)=\frac{A}{L}x,$$ (6)

and so $U_{\text{open}}=\frac{DA}{L}\pi a^{2}$, where *a* is the radius of the tracheal tube.

During the flutter phase, we suppose that the spiracle randomly opens and closes, where the duration of each open or closed bout is an independent exponential random variable. In order to relate our formula (1) to experimental data, we introduce two new parameters. Let *d*_o_ and *d*_c_ denote the average open and closed durations during the flutter phase, respectively. These average durations are related to *p* and *r* by

$$p=\frac{d_{\text{o}}}{d_{\text{o}}+d_{\text{c}}}\;\text{and}\;r=\frac{d_{\text{o}}+d_{\text{c}}}{d_{\text{o}}d_{\text{c}}}.$$ (7)

The mathematical analysis in [19] proves that the steady-state expected value of the solution to the randomly switching partial differential equation (2)–(5) is the linear function

$$c_{\text{flutter}}\left(x\right)=f\frac{A}{L}x=fc_{\text{open}}\left(x\right),$$ (8)

where *f* is given in Eq (1). Multiplying the derivative of this function at *x* = 0 by the diffusion coefficient and the cross-sectional area of the tracheal tube gives the average oxygen uptake during the flutter phase, namely $U_{\text{flutter}}=D\pi a^{2}\frac{d}{dx}c_{\text{flutter}}\left(0\right)=fU_{\text{open}}$.

We used MatLab (The MathWorks, Natick, MA) to evaluate the flutter factor (Eq (1)) for different values of *p*, *D*, *L*, and *r*. We used MatLab and SigmaPlot (Systat software, San Jose, CA) to prepare the figures.

## 3 Results

### 3.1 Dependence of the flutter factor on parameters

The formula for the flutter factor (1) shows that *f* depends on two non-dimensional parameters, the proportion of time open, *p*, and *s* = *rL*^2^/*D*. Notice that the units of *r* are inverse time and the units of *L*^2^/*D* are time, which is the diffusion time from the spiracle to the tracheole. The flutter factor *f* in (1) can be written in terms of *p* and *s*,

$$f\;=\;f\left(p,s\right)\;=\;{\left[1+(\frac{1-p}{p})\frac{\text{tanh}(\sqrt{s})}{\sqrt{s}}\right]}^{-1}.$$

To see the limit of *f* for large *s*, notice that $\text{tanh}(\sqrt{s})$ converges to 1 as *s* goes to infinity, and thus

$$\underset{s\to \infty }{\text{lim}}f\left(p,s\right)=1.$$ (9)

This limit holds for any value of *p*. Therefore, by making *s* large, the insect can make the flutter factor *f* close to 1. Since *U*_flutter_ = *fU*_open_, this means that by raising *s*, the insect can make the rate of oxygen uptake during the flutter phase close to what the oxygen uptake would be if the spiracle were always open. This is true no matter what the value of *p*. This is the main result of the paper.

The parameter *s* = *rL*^2^/*D*. *D* is the diffusion constant of oxygen, which is fixed. *L* is the tracheal length, which will vary between species, but is fixed for a particular species (except during development). Therefore, the adult insect can control the size of *s*, and therefore the flutter factor *f*, by controlling *r*, the rate of fluttering.

Next, we use the fact that lim_*x*→0_tanh(*x*)/*x* = 1. Thus, as *s* goes to zero,

$$\underset{s\to 0}{\text{lim}}f\left(p,s\right)=p,$$

which means that when *r* is small, *U*_flutter_ is approximately *pU*_open_. Therefore, by changing the rate of fluttering, the insect can vary its oxygen uptake between *pU*_open_ and *U*_open_.

The ways in which *f* depends on the other parameters can be seen by straightforward calculations. *f* is a strictly increasing function of *p*, which makes sense because *p* is the percentage of time that the spiracle is open. Further, *f* is a strictly increasing function of *s*, and therefore *f* increases with *r* and *L* and decreases with *D*.

### 3.2 Intuition

The fact that increasing the flutter rate increases the rate of oxygen uptake is a consequence of formula (1), which required a complicated mathematical derivation [19]. We give here a simple intuitive explanation.

In our model, if the concentration in the trachea is *c*(*x*, *t*), then the rate of oxygen uptake at the tracheole is $\pi a^{2}D\frac{\partial c}{\partial x}\left(0,t\right)$. Since *a* and *D* are fixed, the uptake is determined by $\frac{\partial c}{\partial x}\left(0,t\right)$, the slope at *x* = 0. In the special case that the spiracle is always open, *c*_open_(*x*) = *Ax*/*L* and the rate of oxygen uptake is *U*_open_ = *πa*^2^ *DA*/*L*.

**Fig 1:**
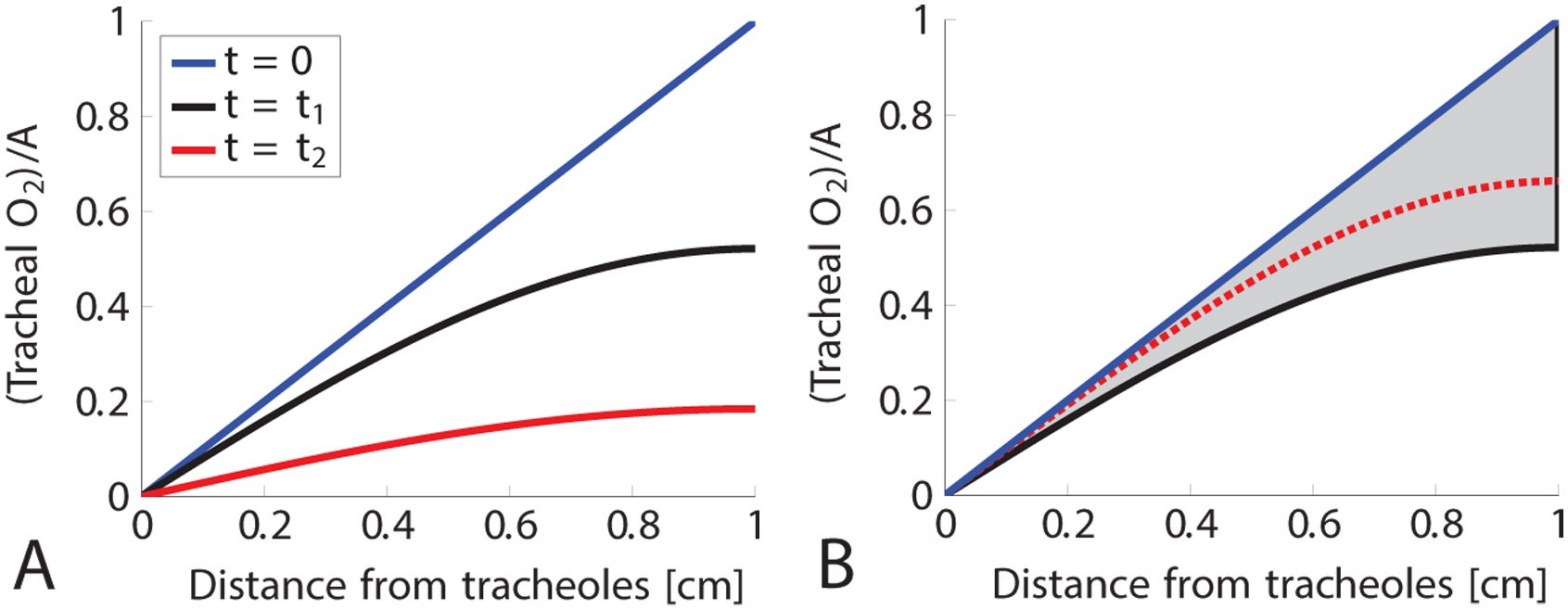
Oxygen profiles at different times. In panel A, the blue curve gives the oxygen profile in the trachea after the spiracle has been open a long time. The spiracle closes at *t* = 0, and the black curve gives the oxygen profile a short time, *t*_1_, later computed from the mathematical model. Notice that the slope of the blue curve and the slope of the black curve at *x* = 0 are essentially the same, and therefore oxygen uptake at the tracheole hasn’t changed much even though the spiracle is closed. Leaving the spiracle closed until a later time, *t*_2_, the oxygen profile is then given by the red curve. The slope of the red curve at *x* = 0 has now decreased significantly which yields a considerable loss in oxygen uptake. In panel B, the blue curve gives the oxygen profile in the trachea after the spiracle has been open a long time, and the black curve gives the oxygen profile after the spiracle has been closed for a short time. If the spiracle opens and closes quickly, then the oxygen profile will stay in the gray region, with a typical profile given by the red dotted curve when the spiracle is closed. Notice that for any such curve, the slope at *x* = 0 is similar to the slope of the blue curve at *x* = 0. Therefore, the rate of oxygen uptake at the tracheole remains high if the spiracle opens and closes quickly.

The blue curve in Fig 1A shows the oxygen concentration after the spiracle has been open for a long time. A short time, *t*_1_, after the spiracle closes, the oxygen concentration is given by the black curve. Note that the slope of the black curve differs very little from the slope the blue curve at *x* = 0, so the oxygen uptake has not changed much. If the spiracle remains closed until a much later time *t*_2_, then the oxygen concentration is given by the red curve. The slope of the red curve at *x* = 0 is much smaller than the slope of the blue and black curves, so by this time the oxygen uptake at the tracheole has dropped considerably. Fig 1B shows why oxygen uptake decreases only slightly if the insect switches quickly between open and closed. After the spiracle closes, the concentration curve has only dropped modestly (black curve) when the spiracle reopens. Therefore the concentration curve will tend to lie between the blue and black curves, and thus the slope at *x* = 0 will not differ very much from the slope of the blue curve.

### 3.3 Respiratory gain

As we have described above, the rate of oxygen uptake is *fU*_open_ in the flutter phase where *U*_open_ is the rate of oxygen uptake in the permanently open steady state. And, if the flutter rate is extremely small the rate of oxygen uptake is *pU*_open_. Thus it is natural to define the **respiratory gain** due to fluttering as:

$$G\;=\;G\left(r,p,L,D\right)\;=\;\frac{fU_{\text{open}}}{pU_{\text{open}}}\;=\;\frac{f}{p}.$$

**Fig 2:**
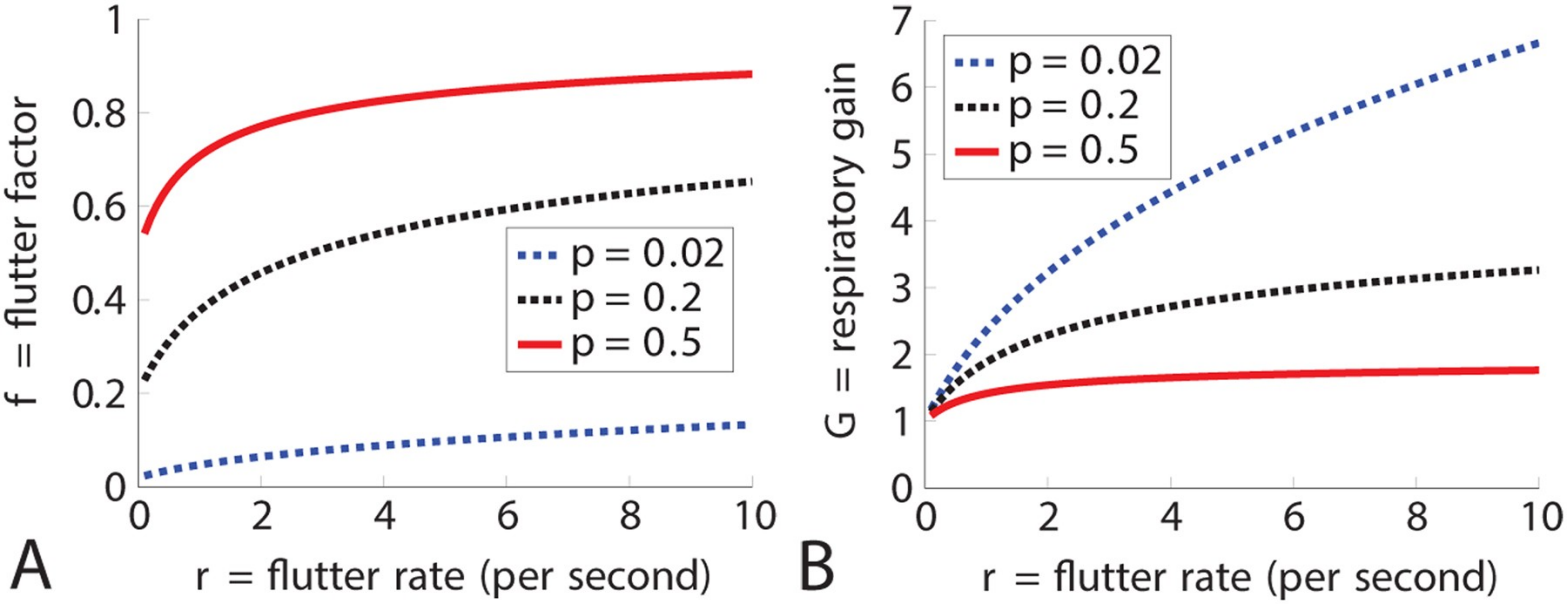
Respiratory gain and the flutter factor for different values of *p*. Panel A shows the flutter factor as a function of flutter rate for three different values of *p*; *p* = 0.02 (blue dashed), *p* = 0.2 (black dotted), and *p* = 0.5 (red solig). For very low flutter rates the flutter factor approaches *p*. Panel B shows the respiratory gain, *G*, in all three cases as a function of flutter rate. The gain can be very high if *p* is small (blue) and is minimal if *p* is large (red). Here, *L* = 1cm and *D* = 0.176cm^2^/sec.

In Panel A of Fig 2, the flutter factor is graphed as a function of flutter rate for three different values of *p*, the proportion of time open in the flutter phase. In each case, as the flutter rate becomes smaller and smaller the flutter factor approaches *p* and as the flutter rate becomes higher that flutter factor rises. Panel B shows the respiratory gain as a function of flutter rate. As the flutter rate increases, so does the respiratory gain. The respiratory gain for the black curve is modest because *p* = 0.5, and the flutter factor is always ≤ 1. The respiratory gain is large for the blue curve because the spiracle is open only 2% of the time so there is an enormous increase in oxygen uptake caused by fluttering.

### 3.4 Specific insects

In the following we discuss the parameters and gain for specific insects. In all cases we take the diffusion constant *D* = 0.176cm^2^/sec [30].

In 1960, Schneiderman conducted experiments on the giant Saturniid silkworms *Hyalophora cecropia*, *Antheraea polyphemus*, and *Samia cynthia* [7]. We take *L* = 1cm. Schneiderman reported that there were “volleys” in which the opening were “commonly less than a second” (we assume 0.5 seconds) and that the openings were separated by 2 to 10 seconds (we assume 5 seconds). This means that *p* = 0.5/5.5 = 0.09. As indicated in (7), if *d*_o_ and *d*_c_ are the open and closed durations, then $r=\frac{d_{\text{o}}+d_{\text{c}}}{d_{\text{o}}d_{\text{c}}}$, so in this case *r* = 2.2sec^−1^. Then, the gain *G* = 2.9, which means that the silkworm increases its oxygen uptake 2.9 fold by fluttering.

In 1993, Lighton studied *Cataglyphis bicolor* and found that fluttering was very rapid, approximately *r* = 24 flutters/sec. Probably that is because it lives in arid conditions and so has to control water loss and because it is small. For the abdominal spiracles we estimate that *L* = 0.28cm. There is no information in the paper on *p*, so we assume *p* = 0.2. Then the respiratory gain is *G* = 2.3. For the thoracic spiracles we estimate that *L* = 0.38cm because of the distance from the spiracles to the head. In that case, the respiratory gain is *G* = 2.6. So the oxygen uptake in this insect benefits enormously from fluttering.

In 2013, Heinrich studied *Gromphadorhina portentosa* and found that the open duration was 0.6 seconds and the closed duration was 1.5 seconds [28]. This gives *p* = 0.28 and *r* = 2.33sec^−1^ by the calculation method in our discussion of *Hyalophora cecropia*. We estimated *L* = 1.6cm. In this case, the respiratory gain is *G* = 2.5.

Sibul et al. studied discontinuous respiration in the pine weevil (*Hylobius abietis*) and found that during the flutter phase spiracles opened and closed about every 10 seconds [22], so *r* = 0.1sec^−1^. *L* is approximately 0.3cm and we took *p* = 0.2. Then *G* = 1.01 so there is almost no respiratory gain from opening and closing at that rate for a beetle of this size.

The last insect that we have discussed shows very little respiratory gain from fluttering because the rate of opening and closing is very low. Note, however, that since this paper did not state the proportion of time open during the flutter phase, we assumed that it was 0.2. If this proportion is much smaller, then the respiratory gain could be significant. Indeed, Fig 6B in [22] suggests that the open durations in the flutter phase may be very small. This reemphasizes the need to obtain accurate data on the proportion of time open during flutter phase. We note, in addition, that Hetz and colleagues [16, 31] caution that the flutter phase is best determined by direct observation, rather than estimation from flow-through respirometry.

## 4 Water loss

In this section, we formulate a mathematical model of water loss from the trachea during the flutter phase. The mathematical analysis of this model is complicated and will be the subject of a subsequent publication [32]. Here we present the model and summarize the main results. In particular, we show that under conditions in which water loss poses a significant threat to the insect, the water loss during the flutter phase is proportional to *p*, the proportion of time the spiracle is open, and is almost independent of the flutter rate *r*. Thus, by having small *p* and large *r*, an insect can both conserve water and have a high oxygen uptake.

Analogous to our oxygen model in section 2, let *w*(*x*, *t*) denote the concentration of water vapor in the trachea at *x* at time *t*. We suppose *w*(*x*, *t*) satisfies the reaction diffusion equation,

$$\begin{array}{cc} \frac{\partial w}{\partial t}\left(x,t\right) & =D_{\text{w}}\frac{\partial^{2}w}{\partial x^{2}}\left(x,t\right)+k\left(I-w\left(x,t\right)\right),\;x\in \left(0,L\right),\;t>0,\\ \frac{\partial w}{\partial x}\left(0,t\right) & =0,\\ w(L,t) & =A_{\text{w}},\;\text{when}\;\text{spiracle}\;\text{open,}\\ \frac{\partial w}{\partial x}\left(L,t\right) & =0,\;\text{when}\;\text{spiracle}\;\text{closed}. \end{array}$$ (10)

Here, *D*_w_ denotes the diffusion coefficient of water and *A*_w_ denotes the ambient water concentration. The key difference between this model and the oxygen model is the term *k*(*I*−*w*(*x*, *t*)) in (10) which models transfer of water into the trachea through the walls of the trachea. The parameter *k* is the water transfer rate and *I* is the equilibrium water concentration in the trachea if the spiracle is always closed. If the spiracle is always open, then the steady state water loss per unit time is

$$W_{\text{open}}=\left(I-A_{\text{w}}\right)\pi a^{2}\sqrt{kD_{\text{w}}}\text{tanh}\left(\sqrt{kL^{2}/D_{\text{w}}}\right),$$ (11)

where *a* is the radius of the tracheal tube. The formula (11) comes from setting the right hand side of (10) equal to zero and solving for the steady state concentration *w*(*x*) when the spiracle is always open. The rate of water loss is then $D_{\text{w}}\frac{dw}{dx}\left(L\right).$.

As in our oxygen model, we suppose that the spiracle randomly opens and closes during the flutter phase with parameters *p* and *r*. If *W*_flutter_ denotes the water loss per unit time during the flutter phase, we define the *water flutter factor* to be the ratio, *f*_w_ = *W*_flutter_/*W*_open_. In a subsequent paper [32], after a lengthy mathematical derivation, we obtain the following formula for *f*_w_,

$$\begin{array}{c} f_{\text{w}}\left(p,\sigma ,\kappa \right)\\ \;=\frac{\frac{1}{4}\left(e^{2\sqrt{\kappa }}+1\right)pe^{-\sqrt{\kappa +\sigma }-\sqrt{\kappa }}\left(e^{2\sqrt{\kappa +\sigma }}-1\right)\left(\kappa +\sigma \right)}{p\left(\kappa +\sigma \right)\text{cosh}\left(\sqrt{\kappa }\right)\text{sinh}\left(\sqrt{\kappa +\sigma }\right)+\left(1-p\right)\sqrt{\kappa (\kappa +\sigma )}\text{sinh}\left(\sqrt{\kappa }\right)\text{cosh}\left(\sqrt{\kappa +\sigma }\right)}, \end{array}$$

where *σ* = *rL*^2^/*D*_w_ and *κ* = *kL*^2^/*D*_w_ are dimensionless parameters describing how the flutter rate and water transfer rate compare to the diffusion time. Notice that *f*_w_ is independent of *I* and *A*_w_. That doesn’t mean that water loss is independent of *I* and *A*_w_, just that the percent change due to fluttering compared to being always open is independent. The flutter factor, *f*_w_, does depend on *p*, *σ*, and *κ*, and the first two can be estimated from the values of *p*, *r*, and *L* for various insects in section 3.4 above (and we take *D*_w_ = 0.282cm^2^/sec [30]). Thus, the only remaining undetermined parameter is *k*.

**Fig 3:**
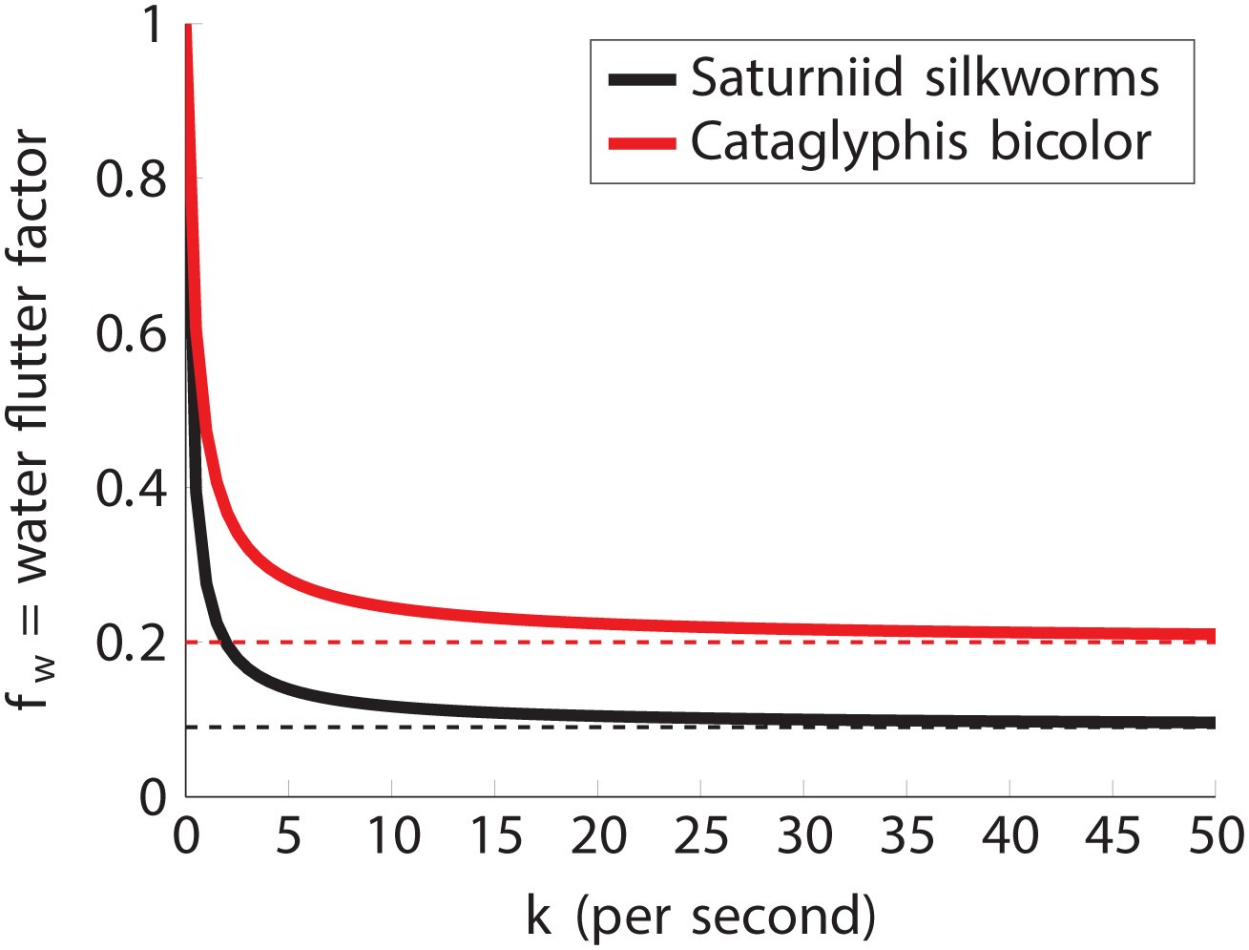
Water flutter factor *f*_w_ as a function of water transfer rate *k* for two different insects. The black curves correspond to the giant Saturniid silkworms whose parameters *p*, *f*, and *L* were estimated in section 3. Similarly, the red curves are for *Cataglyphis bicolor*. For both black and red, the solid curves are *f*_w_ and the dashed lines are *p*. For *k* small, there is little water loss (see (11)) and for *k* large *f*_w_ is approximately *p* so the water loss is proportional to the percentage of time the spiracle is open independent of flutter rate. Thus, the insect can achieve both low water loss (low *p*) and high oxygen uptake (high *r*) simultaneously.

Unfortunately, the water transfer rate *k* is difficult to estimate. However, it follows immediately from (11) that if *k* is small, then the water loss is also small. Hence, water loss poses a threat to the insect only for sufficiently large *k*. In Fig 3, we plot *f*_w_ as a function of *k* for two of the insects studied in section 3.4 above. This figure illustrates that *f*_w_ rapidly approaches *p* as *k* increases. In fact, one can use the explicit formula for *f*_w_ to show that

$$\underset{\kappa \to \infty }{\text{lim}}f_{\text{w}}=p,\;\text{where}\;\kappa =kL^{2}/D_{\text{w}}.$$ (12)

Therefore, Fig 3 and Eq (12) show that if *k* is large, then the water loss during the flutter phase is approximately proportional to *p*, the percentage of time the spiracle is open. Hence, in the case that water loss threatens the insect (large *k*), it can conserve water by keeping its spiracles closed most of the time (small *p*). Our results in section 3 show that the insect can simultaneously maintain a high oxygen uptake by fluttering rapidly (large *r*).

## 5 Discussion

**Fig 4:**
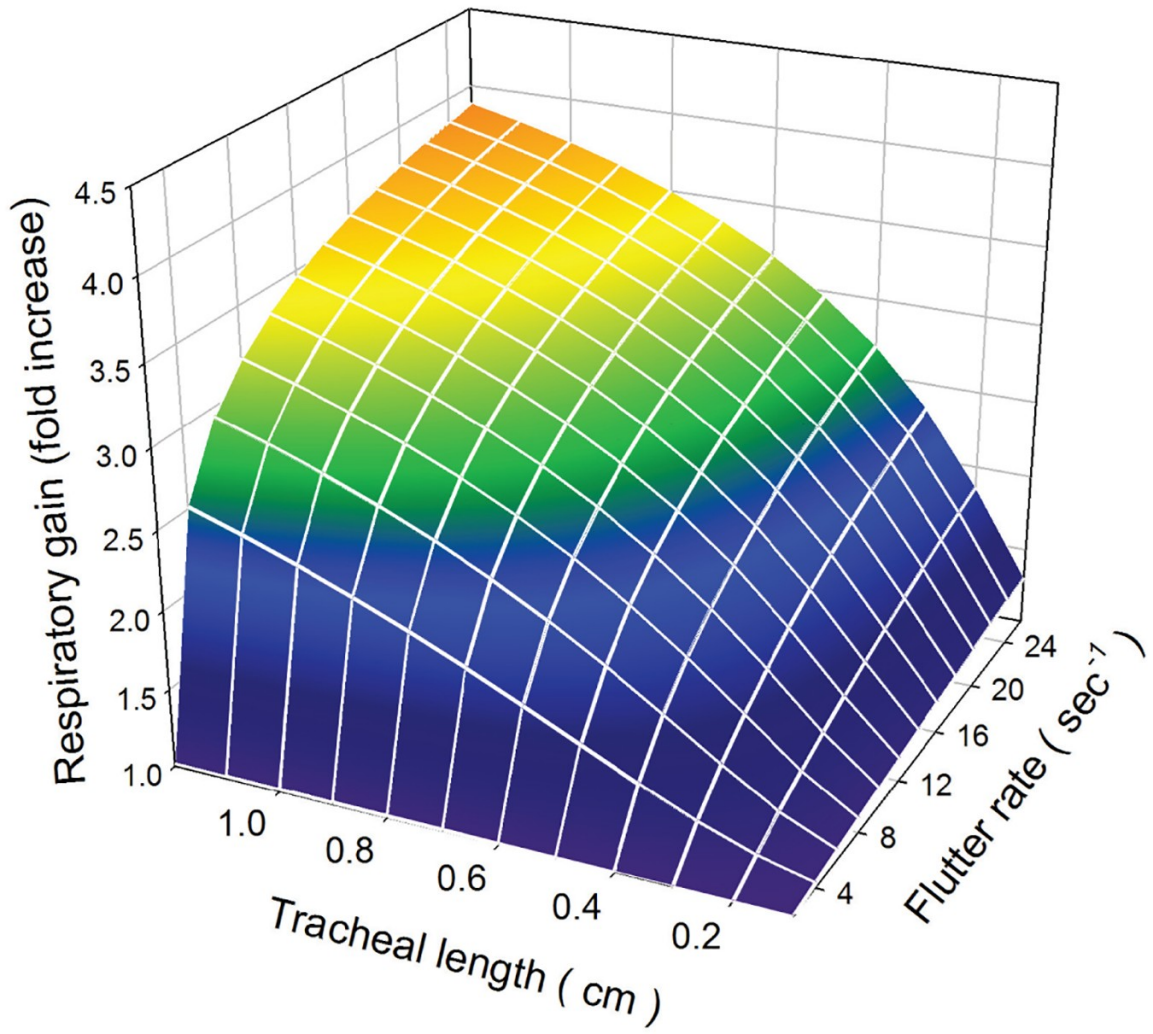
Respiratory gain as a function of tracheal length and flutter rate. We assume that *p* = 0.2. Increasing either tracheal length or flutter rate increases respiratory gain. The gains would be even larger if *p* were smaller.

Our mathematical calculations show that the oxygen uptake during the flutter phase can be increased by increasing the rate of fluttering, *r*. The respiratory gain (fold change compared to not fluttering) depends on *p*, the proportion of time open, *L*, the length of the trachea, and *r*, the flutter rate. Thus, the actual gain will vary from insect to insect. Fig 4 shows the respiratory gain as a function of *r* and *L*. The figure shows that the larger an insect is, the more it gains by increasing its flutter frequency. Insects with a tracheal length of less than 1 millimeter gain very little by even fairly rapid fluttering. Of course, small insects have a very short diffusion distance and so probably do not need mechanisms to enhance oxygen uptake. Small insects could achieve a large respiratory gain by extremely rapid fluttering, but that would be mechanistically and energetically unfeasible. We note that a number of studies have investigated how the flutter phase scales scales with mass [8, 33–36].

Since the insect can control the rate of fluttering, *r*, it can control oxygen uptake. As we have indicated, the oxygen uptake *U*_flutter_ = *fU*_open_ and the insect can change *f* by changing the rate *r* of opening and closing. *f* will vary from *p* for very low rates to almost 1 for high rates. As indicated in Table I of [7], the measured percentage of time spent fluttering varies widely among silkworms, varying from 39% to 91%, consistent with the idea that fluttering delivers more oxygen uptake. In Table II of [7], it is indicated that flutter frequency increases with day of development, which is what we would expect since the metabolic rate of pupae during adult development (in the pupae) increases and the insect can increase the required oxygen uptake to meet this need by increasing the flutter frequency.

We have derived a formula for the average oxygen uptake per unit time during the flutter phase, *fU*_open_, so it is straightforward to derive a formula for the average oxygen uptake per unit time over the entire discontinuous gas-exchange cycle. Let *p*_f_, *p*_c_, and *p*_o_ be the percentage of time that the insect spends in the flutter, closed, and open phases, respectively. We stress that *p*_o_ should not be confused with the parameter *p* used in Eq (1) since *p* is the percentage of time the spiracles are open *within* the flutter phase. The average oxygen uptake per unit time over the entire discontinuous gas-exchange cycle is therefore

$$p_{\text{f}}U_{\text{flutter}}+p_{\text{c}}U_{\text{closed}}+p_{\text{o}}U_{\text{open}}=(fp_{\text{f}}+p_{\text{o}})U_{\text{open}},$$ (13)

since the uptake in the closed phase *U*_closed_ is approximately zero. Eq (13) reveals that an insect can control its oxygen uptake through fluttering in two distinct ways: it can regulate the percentage of time it spends fluttering, *p*_f_, and it can regulate the flutter rate, *r*, within the flutter phase which thereby regulates *f*. Note that either control mechanism allows the insect to keep its spiracles closed most of the time.

The mathematical model given in (2)–(5) is very simple in that it does not include convection, which is known to be important, and it has very simple boundary conditions at *x* = 0 and *x* = *L*. In the Appendix, we formulate a more general model that includes convection and derive a (more complicated) formula for the flutter factor *f*(*p*, *r*, *L*, *D*, *v*). When the convection velocity *v* = 0, this *f* reduces to formula (1). Furthermore, as we show in the Appendix, *f*(*p*, *r*, *L*, *D*, *v*) → 1 for high flutter rates. Therefore, the main result of this paper is true even when convection is included.

No mathematical model can capture the full complexity of physiological systems. For example, we have assumed that oxygen diffuses freely in the trachea, but this approximation may not be good for extremely narrow tracheal tubes [37]. It is reasonable to include more physiological boundary conditions at *x* = 0 and *x* = *L* in the model. Berezhkovskii and Shvartsman [38] developed a mathematical model generalizing our work in [19] that included a boundary condition at *x* = *L* corresponding to the fact that the spiracle has a finite radius *a*. They derived a formula for the flutter factor that reduces to Eq (1) in the case that the tracheal length is much larger than the tracheal radius, which is true for most insects. In our model, the boundary condition at *x* = 0 corresponds to the tracheole being perfectly absorbing. If instead, we use a boundary condition that corresponds to partial uptake, we can derive a formula for the flutter factor (not shown) and it approaches 1 for fast flutter rates. So, the basic result of this paper holds for physiological models with more complicated boundary conditions.

It is clear from the experimental literature that water loss is a serious problem. When insects are forced to keep their spiracles open, their rate of water loss increases 5-10 fold [1]. The bug *Rhodnius* dies from dehydration in about 3 days if it is forced to keep its spiracles open, whereas it can survive for weeks to months if it is allowed to regulate its spiracle closure [2]. It is natural to ask how water loss is affected by fluttering. In Section 4, we presented a mathematical for water loss in the presence of fluttering that includes water entry into the tracheal tube and efflux through the spiracle. The mathematical analysis of the model is difficult and will appear in [32]. In Section 4 we show the relevant results. When the transfer coefficient, *k*, from tissue to the tracheole is small there is little water loss and when the transfer coefficient is large the water loss is proportional to *p*, almost independent of the flutter rate. Thus, by having small *p* and large *r*, an insect can both conserve water and have a high oxygen uptake, effectively decoupling the challenge of preventing water loss from the challenge of obtaining adequate oxygen.

However, our work gives only a partial understanding of the interesting and difficult issues involved in discontinuous gas exchange in insects. First, fluttering is clearly energetically expensive. Second is the problem of how insects get rid of *CO*_2_. This gas is much more soluble in water than *O*_2_ and is thus trapped in cells causing the tracheal pressure to be lower than ambient pressure. When the spiracle opens, this pressure difference causes inward convective flow which aids *O*_2_ uptake but hinders *CO*_2_ release. Woods and Smith have created a very general model for water and gas exchange with the environment and have shown that water loss scales proportionally to *O*_2_ (or *CO*_2_) uptake in mammals, insects, birds, bird eggs, and plants [39, 40]. Of course, individual species may have developed special mechanisms (like fluttering or kidneys) that help them solve the problem of balancing water loss against *O*_2_ uptake. But, as Woods and Smith point out, *CO*_2_ release may be the more difficult issue for insects and may require them to keep their spiracles open much longer than needed for *O*_2_ uptake leading to much more water loss. Thus, a fuller understanding of discontinuous gas exchange would require the inclusion of *CO*_2_ storage in cells and exchange with the trachea in our model, which will be the subject of future work.

The respiratory system of insects has to solve various physiological challenges. The insects must match oxygen uptake to metabolic needs. They must release *CO*_2_ at the average rate at which it is produced and they must do this while minimizing water loss from the tracheal system. They must balance the advantages of increased body size with the concomitant disadvantage of increased diffusion length. And they must solve these challenges with mechanisms that are not energetically costly. The right solutions to these distinct challenges are likely to vary considerably since insects differ greatly in size, anatomy, and physiology, and are adapted to live in very different environments [41, 42]. What we have shown is that variation in flutter rate is an effective mechanism for controlling oxygen uptake, and therefore is an important part of insects’ solutions to these anatomical, physiological, and environmental challenges.

## Appendix

When the spiracle is closed, the pressure in the tracheal tube drops below ambient pressure because oxygen is removed to the tissues faster than it is replaced by carbon dioxide coming from the tissues. The reason is that carbon dioxide is more soluble in water than oxygen and the carbon dioxide binds to proteins in the cells [8, 23, 24]. The result is that when the spiracle opens there is a net inward flow. In this Appendix, we generalize our model in (2)–(5) to include convection. The oxygen concentration *c*(*x*, *t*) now satisfies

$$\frac{\partial c}{\partial t}\left(x,t\right)=D\frac{\partial^{2}c}{\partial x^{2}}\left(x,t\right)-v\frac{\partial c}{\partial x}\left(x,t\right),\;x\in \left(0,L\right),\;t>0.$$ (14)

Here, *v* < 0 is the convection velocity corresponding to inward bulk flow. The boundary condition at *x* = 0 in (3), and the boundary condition at *x* = *L* when the spiracle is open in (4) are unchanged. The no flux boundary condition at *x* = *L* when the spiracle is closed is now

$$-D\frac{\partial c}{\partial x}\left(L,t\right)+vc\left(L,t\right)=0,\;\text{when}\;\text{spiracle}\;\text{closed}.$$

Applying the mathematical techniques that we developed in [43] and [44], the large time expected value of the solution to this randomly switching partial differential equation is given by ${\text{lim}}_{t\to \infty }E\left[c\left(x,t\right)\right]=u_{0}\left(x\right)+u_{1}\left(x\right)$, where *u*_0_ and *u*_1_ satisfy the boundary value problem

$$\begin{array}{cc} 0 & =Du_{0}^{\prime \prime }-vu_{0}^{\prime }-\left(1-p\right)ru_{0}+pr_{1},\;x\in \left(0,L\right),\\ 0 & =Du_{1}^{\prime \prime }-vu_{1}^{\prime }+\left(1-p\right)ru_{0}-pr_{1},\;x\in \left(0,L\right),\\ u_{0}\left(0\right) & =u_{1}\left(0\right)=0,\;u_{0}\left(L\right)=pA,\;-Du_{1}^{\prime }\left(L\right)+vu_{1}\left(L\right)=0. \end{array}$$ (15)

The steady-state expected oxygen uptake during the flutter phase is given by

$$U_{\text{flutter}}=\pi a^{2}[D\frac{\partial }{\partial x}u_{0}\left(0\right)+D\frac{\partial }{\partial x}u_{1}\left(0\right)-v\left(u_{0}\left(0\right)+u_{1}\left(0\right)\right)].$$

The steady-state oxygen uptake if the spiracle is always open can be found by setting *p* = 1 in (15) and is given by

$$U_{\text{open}}=\frac{\pi a^{2}Av}{1-e^{vL/D}}.$$

It is straightforward to solve (15) and find the flutter factor

$$f=\frac{U_{\text{flutter}}}{U_{\text{open}}}=\frac{p\left(e^{V}-1\right)[V(-e^{\sqrt{4s+V^{2}}})+(e^{\sqrt{4s+V^{2}}}+1)\sqrt{4s+V^{2}}+V]}{p(e^{V}-1)(e^{\sqrt{4s+V^{2}}}+1)\sqrt{4s+V^{2}}-V(pe^{V}+p-2)(e^{\sqrt{4s+V^{2}}}-1)},$$ (16)

where *s* = *rL*^2^/*D* and *V* = *vL*/*D*. Note that if we take *V* → 0, then (16) reduces to (1). Taking the fast fluttering limit of (16) yields

$$\underset{s\to \infty }{\text{lim}}f=1.$$ (17)

This limit as *s* → ∞ is analogous to the limit in Eq (9) for the model without convection. Thus, the main result of this paper, that the insect can increase oxygen uptake by more and more rapid fluttering to get close to the oxygen uptake if the spiracle were always open, holds whether or not convection is included in the model.
